# Strain specific differences in rates of Photosystem II repair in picocyanobacteria correlate to differences in FtsH protein levels and isoform expression patterns

**DOI:** 10.1371/journal.pone.0209115

**Published:** 2018-12-19

**Authors:** Erin M. Bonisteel, Brooke E. Turner, Cole D. Murphy, Jenna-Rose Melanson, Nicole M. Duff, Brian D. Beardsall, Kui Xu, Douglas A. Campbell, Amanda M. Cockshutt

**Affiliations:** 1 Department of Chemistry & Biochemistry, Mount Allison University, Sackville, New Brunswick, Canada; 2 Department of Biology, Mount Allison University, Sackville, New Brunswick, Canada; 3 Environmental Microbiomics Research Center, School of Environmental Science and Engineering, Sun Yat-sen University, Guangzhou, China; University of Hyderabad School of Life Sciences, INDIA

## Abstract

Picocyanobacteria are the numerically dominant photoautotrophs of the oligotrophic regions of Earth’s oceans. These organisms are characterized by their small size and highly reduced genomes. Strains partition to different light intensity and nutrient level niches, with differing photosynthetic apparatus stoichiometry, light harvesting machinery and susceptibility to photoinactivation. In this study, we grew three strains of picocyanobacteria: the low light, high nutrient strain *Prochlorococcus marinus* MIT 9313; the high light, low nutrient *Prochlorococcus marinus* MED 4; and the high light, high nutrient marine *Synechococcus* strain WH 8102; under low and high growth light levels. We then performed matched photophysiology, protein and transcript analyses. The strains differ significantly in their rates of Photosystem II repair under high light and in their capacity to remove the PsbA protein as the first step in the Photosystem II repair process. Notably, all strains remove the PsbD subunit at the same rate that they remove PsbA. When grown under low light, MIT 9313 loses active Photosystem II quickly when shifted to high light, but has no measurable capacity to remove PsbA. MED 4 and WH 8102 show less rapid loss of Photosystem II and considerable capacity to remove PsbA. MIT 9313 has less of the FtsH protease thought to be responsible for the removal of PsbA in other cyanobacteria. Furthermore, by transcript analysis the predominant FtsH isoform expressed in MIT 9313 is homologous to the FtsH 4 isoform characterized in the model strain *Synechocystis* PCC 6803, rather than the FtsH 2 and 3 isoforms thought to be responsible for PsbA degradation. MED 4 on the other hand shows high light inducible expression of the isoforms homologous to FtsH 2 and 3, consistent with its faster rate of PsbA removal. MIT 9313 has adapted to its low light environment by diverting resources away from Photosystem II content and repair.

## Introduction

Picocyanobacteria, comprised of marine *Synechococcus* and *Prochlorococcus*, are the numerically dominant group of phytoplankton performing oxygenic photosynthesis in the oligotrophic regions of Earth’s oceans [1], together contributing between 32 and 80% of oceanic primary productivity [2–6]. With the expected increase in stratification resulting from increasing sea temperatures, it has been predicted that the global distribution of the picocyanobacteria will expand [7–9]. While *Prochlorococcus* dominates in the more oligotrophic waters from 40°S to 40°N and grows poorly in cooler waters [10–13], marine *Synechococcus* tolerates a broader temperature range and exploits more meso- and eutrophic water [13,14]. *Synechococcus* and *Prochlorococcus* each have different cell types, commonly referred to as ecotypes, characterized by different temperature, nutrient and light regime preferences [15]. While *Synechococcus* ecotypes, which often differ in their phycobilisome light harvesting pigment complexes, occupy different niches along a horizontal onshore-offshore axis [16,17], *Prochlorococcus* is distributed along a vertical depth axis, from high light at the surface to much lower light levels, but generally higher nutrient levels, at the very bottom of the euphotic zone [15].

*Prochlorococcus* and marine *Synechococcus* are thought to have evolved from a shared ancestor 100–200 million years ago [18]. *Prochlorococcus* ecotypes are characterized by extensive genome streamlining and very small cell size, adaptations that are thought to impart competitive advantages for survival in nutrient limited environments (reviewed in [19]). A major difference between the two genera is the loss from *Prochlorococcus* of the genes encoding the nitrogen rich phycobilisomes responsible for light harvesting in *Synechococcus* [20]. Instead of phycobilisomes, *Prochlorococcus* have Prochlorophyte chlorophyll binding proteins (Pcb) that are homologous to the low iron inducible Iron Stress Inducible (IsiA) proteins that can act as light harvesting proteins for Photosystem I (PSI) in cyanobacteria grown under iron limitation [20]. While the light harvesting machinery for photosynthesis is different for these two genera, the remainder of the genes encoding the photosynthetic apparatus are highly conserved [21]. Zorz et al [22] examined the photophysiology and photosynthetic protein stoichiometry in three strains of picocyanobacteria: the high light, high nutrient *Synechococcus* WH 8102; the high light, low nutrient *Prochlorococcus* MED 4; and the low light, high nutrient *Prochlorococcus* MIT 9313. They showed that when grown under similar conditions of light and temperature, MIT 9313 has a much higher ratio of PSI:PSII than do MED 4 or WH 8102. In addition to their large pool of PSI, the MIT 9313 cells have little Rubisco compared to the *Synechococcus* strain. In that study, the electron transport rate away from PSII correlated best with the ratio of Rubisco:PSII, suggesting that ultimately the capacity for electron transfer to CO_2_ limits the rate of flow of electrons away from PSII [22]. Partensky et al [23] have recently shown that oxygen evolution rates differ between *Prochlorococcus* and *Synechococcus* strains, with PCC 9511 (equivalent to MED 4) achieving the highest oxygen evolution per PSII, an effect that increased with irradiance, while the low light strains MIT 9313 and SS120 show lower oxygen evolution per PSII that decreased with increased irradiance. These results show that different ecotypes of picocyanobacteria differ significantly in the photosynthetic strategies that they employ to survive in their respective niches.

Photosynthesis exposes cellular components, in particular the proteins of the photosystems, to damage from the light required for the process and from the oxygen evolved. A photon in the blue or UV range absorbed by the Mn_4_Ca cluster of the oxygen evolving complex can lead to inactivation of the PSII centre [24]. With the oxygen evolving complex compromised in this way, the P680^+^ radical cannot be rapidly reduced, leading to oxidative damage of the PsbA core protein of the reaction centre [25,26]. A second inactivation mechanism involves generation of the reactive oxygen species singlet oxygen (^1^O_2_) which happens to a greater extent when the plastoquinone pool taking electrons away from PSII becomes increasingly reduced, increasing the lifetime of the triplet state of P680 (for a review see [26]). Six et al [27] showed that under moderately high light different *Synechococcus* and *Prochlorococcus* strains have similar σ_I_ (effective target size for photoinactivation) values. In a subsequent study of *Synechococcus* WH 8102 and *Prochlorococcus* MED 4 photophysiology, Murphy et al [28] showed that the inherent photoinactivation potential of an incident blue light photon, in the absence of excitation pressure, differed between strains and was dependent on growth light for *Synechococcus*. Furthermore, they showed that the yield of PSII photoinactivations per photon delivered to PSII through the effective absorption cross section for photochemistry fell on a single line for both picocyanobacterial strains and growth lights, with a slope that depended on the excitation pressure on PSII. Soitamo et al [29] examined the photoinactivation of a number of picocyanobacteria at a range of light levels. They showed that low light *Prochlorococcus* strains (SS 120 and MIT 9313) are more sensitive to photoinhibition than a high light *Prochlorococcus* strain, and both of these were more sensitive than the marine *Synechococcus* strain WH 8103.

Regardless of the mechanisms of PSII inactivation, a repair process needs to occur to maintain photosynthesis. The PSII repair cycle (reviewed in [30]) involves partial disassembly of the PSII complex, removal and degradation of damaged PsbA (and possibly other) proteins, insertion of newly synthesized protein(s) and reassembly of the active complex. The rate limiting step of this cycle is likely either the rate of damaged PsbA removal or the rate of synthesis of new replacement protein [31], depending upon taxa and conditions. In model cyanobacterial species such as *Synechocystis* PCC 6803, PsbA removal is thought to be carried out primarily by a heterohexamer of the protease subunits FtsH 2 and 3 (slr0228 and slr1604, respectively) [32,33]. FtsH has a transmembrane region, a AAA^+^ module (ATPase associated with diverse cellular activities) and a Zn^2+^ binding protease domain [34]. FtsH proteins are thought to be able to form both hetero- or homohexamers [33]. The N-terminal region of PsbA is thought to be altered or exposed as a result of damage, which identifies the protein to FtsH for removal [35].

Little is known about the biochemical mechanism of the PSII repair cycle in picocyanobacteria. Six et al [27] reported that while the different picocyanobacterial strains show similar effective target sizes for photoinactivation, they differ greatly in their functional rate constants for PSII repair following photoinactivation, with the *Synechococcus* strains inducing more rapid repair than *Prochlorococcus*, and the high light ecotype of *Prochlorococcus* inducing faster repair than the low light ecotype studied. In that study, the rate constant for repair of PSII in the low light adapted strain, SS120, was almost an order of magnitude slower than that measured for PCC 9511, which is equivalent to MED 4. The biochemical mechanism(s) that underlie this difference in the ability of strains to repair inactivated PSII is not known. In the current study we have therefore combined quantitative protein analyses, photophysiology and transcript analysis to elucidate these differences across ecotypes and growth lights.

## Materials and methods

### Experimental design

For this study we combined parallel analyses of photophysiology and protein determinations. Cultures were grown in flasks and then a volume of 50 mL was concentrated by centrifugation to ~ 3.5 mL volumes and evenly resuspended. This sample was then used for photophysiology measurements. Following the measurements, a 100 μL sample was saved for chlorophyll determination and 2 mL were centrifuged to form a pellet for protein analysis. Parallel cultures were used for transcript analyses.

### Cell culturing

Cultures of *Synechococcus* sp. WH8102, and *Prochlorococcus* MED4 and MIT9313 (all obtained from Bigelow Labs, NCMA Maine, US) were grown in PCR-S11 marine medium [36] and Pro99 medium [37], respectively. The PCR-S11 and Pro99 media were made using filtered, autoclaved sea water. “Mother Cultures” were grown in 125 mL Erlenmeyer flasks, from which “working cultures” were inoculated at an approximately 1 in 5 dilution factor into 500 mL Erlenmeyer flasks. Both high light and low light cultures were grown and inoculated at light intensities of 260 μmol photons m^-2^ s^-1^ and 30 μmol photons m^-2^ s^-1^, respectively, with the exception of *Prochlorococcus* MIT9313 which was grown at 90 μmol photons m^-2^ s^-1^ and 30 μmol photons m^-2^ s^-1^. All cultures were grown at 22°C with 12:12 L:D photoperiod.

To monitor the growth rate of the cells, measurements were taken daily using a Spectra Max Gemini EM Spectrofluorometer (Molecular Devices). The spectrofluorometer generated absorbance spectra measured from 630 nm to 670 nm with a peak at 650 nm for *Synechococcus* and from 660 nm to 700 nm with a peak at 680 nm for *Prochlorococcus*. The raw peak was used as a proxy for pigment content, and thus, cell content. Absorbance vs. time was fit to monitor growth with a modified Gompertz growth equation [38] model that uses lag phase, carrying capacity and growth rate as fitted parameters using the minpack.lm package [39] in RStudio [40]. Cultures were sampled during the exponential growth phase.

### Photophysiology

A volume of 50mL of cell culture was spun down at 4200 rcf for 20 minutes in a JA17 rotor (Beckman Coulter Avanti J-20 centrifuge). The supernatant was then discarded until about 3.5 mL remained, and the pellet was resuspended using a pipettor. This volume of concentrated cell suspension was used immediately for physiology measurements. One hundred μL of this cell suspension was taken for chlorophyll determinations after the physiology measurements.

Detailed methods for physiological measurements and calculations follow [25,28,41]. Briefly [41,42] photoinactivation and repair treatments and measurements were carried out in Photon Systems Instruments FL3500 super-heads (Drasov, Czech Republic) with a lab-built aluminum plug for temperature control. For each treatment, a 2.5 mL sample was loaded into a cuvette with a micro stir-bar and plugged to hold the temperature at 22±0.1°C through circulation of cooling fluid through the aluminum plug. The super-head provided three capacities: firstly, application of sub-saturating flashlets of blue light of 1.2 μs duration in blue (455 nm LED); secondly, detection of the fluorescence emission resulting from the flashlets; and thirdly, delivery of a sequential series of 10 periods of 330 s of actinic blue light at 0, 30, {8 x 260} μmol photons m^-2^-s^-1^. The cells were thus cumulatively exposed to 2640 s of moderately high light during the treatment/measurement protocol. To measure photoinactivation and the counteracting repair lincomycin at a final concentration of 500 μ g mL^-1^, was added, or not, to replicate cuvettes to inhibit prokaryotic ribosomes and thereby block the PSII repair cycle during the light treatments [41,42].

We used the super-head flashlet capacity to perform fast repetition rate (FRR) chlorophyll fluorescence induction profiles driven by a train of 40 flashlets of 1.2 μs each separated by 2.0 μs dark, for a cumulative flashlet train duration of 128 μs. FRR inductions were measured at the end of each treatment period step in the presence of actinic light (if any), and then again 2 s after the end of actinic light exposures.

FRR inductions and relaxations were fit to quantify PSII physiology [43] using the psifluo data handling and fitting package [44] implemented in the RStudio environment [40]. We thereby extracted photophysiological parameters including the effective absorption cross section for PSII photochemistry, σ_PSII_, the minimal, F_0_ʹ, and maximal, F_M_ʹ, fluorescence emission in the light acclimated state, and the lifetimes for re-opening of PSII through electron transport, τ_1_ & τ_2_. We then estimated the apparent maximal quantum yield for PSII photochemistry:

$$\frac{{\text{F}}_{\text{V}}^{\text{'}}}{{\text{F}}_{\text{M}}^{\text{'}}}=\frac{{\text{F}}_{\text{M}}^{\text{'}}-{\text{F}}_{0}^{\text{'}}}{{\text{F}}_{\text{M}}^{\text{'}}}$$

F_V_ʹ /F_M_ʹ values were then plotted against elapsed time for the high light treatment light period. We fit the data measured in the presence of lincomycin with a single phase exponential decay equation, using F_V_ʹ/F_M_ʹ as a proxy for [PSII_active_]:

$$\left[{\text{PSII}}_{\text{active}}]\left(t\right)=[{\text{PSII}}_{\text{active}}\right]\left(t_{0}\right)e^{-k_{\text{PI}}t}$$

where t is elapsed time and k_PI_ is a first order rate constant for photoinactivation of PSII with units of s^-1^.

We then used the F_V_ʹ/F_M_ʹ values captured in the absence of lincomycin to estimate the functional first order rate constant for PSII repair, k_REC_. Using the assumption that k_PI_ remains the same in the absence of PSII repair (+lincomycin) or presence of PSII repair (-lincomycin) for each replicate we took the k_PI_ value fit in the presence of lincomycin as an input into the equation [25,45]:

$$\left[{\text{PSII}}_{\text{active}}]\left(t\right)=[{\text{PSII}}_{\text{active}}\right]\left(t_{0}\right)\frac{k_{\text{REC}}+k_{\text{PI}}e^{-(k_{\text{PI}}+k_{\text{REC}})t}}{k_{\text{PI}}+k_{\text{REC}}}$$

Simultaneously with the chlorophyll fluorescence measures we tracked the concentration of dissolved oxygen in the cell suspensions in the cuvettes using a FireSting optode (PyroScience, Germany) [41]. We followed the protocols of Ni et al [41] to measure oxygen exchange in μmol O_2_ ml^-2^ s^-1^ at 0 μmol photons m^-2^-s^-1^ before any light treatment, at 30 μmol photons m^-2^-s^-1^, and then over the 8 x 330 s periods of 260 μmol photons m^-2^-s^-1^. We then measured dark oxygen exchange after the high light exposure. In addition, both before and then again after the high light time course we used oxygen flash yield measures [46,47] to quantify μmol PSII ml^-2^. Steady state and flash yield oxygen data was assembled, transformed and analyzed using the tidyverse [48] and minpack.lm [39] packages running under RStudio [40].

### Quantitative immunoblotting

For each replicate, 2 mL of the concentrated culture from the physiology cuvette were transferred into a 2 mL microcentrifuge tube for subsequent protein work. Two μL of pluronic acid was added to the cell suspension in the microcentrifuge tube before being spun down in a tabletop centrifuge (Mikro 20 Hettich zentrifugen) for 5 minutes at 13000 rpm. The supernatant was removed and the resulting pellet was flash frozen in liquid nitrogen and stored at -80°C for later use.

Cell pellets were resuspended in 300–500 μL of protein extraction buffer (1X Pefabloc/AEBSF and 1X PSB). The resuspended material was transferred to a 2 mL ceramic bead Lysing Matrix Tube “Lysing Matrix D” (MPBIO), and subsequently homogenized using the CY:24x2 rotor of the FastPrep-24 Instrument (MPBIO) at 6.5 m/s for 60 seconds, followed by a minute of cooling on ice and repeated a total of 3 times. The samples were centrifuged for 5 minutes at 14,800 x *g* and the supernatant was collected and transferred to a 0.6 mL microtube. The extracted protein samples were aliquoted in ~40 μL portions and stored in a -80°C freezer to avoid repeated freeze thaw cycles.

Protein concentrations of whole cell extracts were measured using the BCA Protein Assay Kit (ThermoFischer Scientific, Cat. 23225) using the microplate procedure with bovine gamma globulin (BGG; Bio-Rad) for protein standards ranging from 0.0 to 1.0 mg/mL. Samples were diluted with IX PSB as required. A SpectraMax microplate reader (Molecular Devices) was used to measure absorbance at 562 nm. Protein concentrations were measured in triplicate for each sample, and these concentrations were used to determine protein loads for quantitative immunoblotting.

Immunoblotting was performed as described in [49]. 4–12% acrylamide 17-well gels (Bolt Bis-Tris Plus gels, Invitrogen) in MES SDS running buffer (Bolt, Invitrogen) in Bolt Mini Gel Tanks (Invitrogen) were used in all electrophoresis protocols. All protein samples were made to a concentration of 1X LDS Sample Buffer (4X stock, Life Technologies) and 50 mM dithiothreitol (DTT). The protein samples were heated to 70°C for 5 minutes and spun briefly. Protein loads of 0.25 to 4.0 μg were used, and standards were loaded in ranges from 7 to 250 fmoles per load (Agrisera). Samples were separated by electrophoresis for 20–35 minutes at 200V (depending on protein size). The proteins were then transferred via electrophoresis from the gel to a PVDF membrane using Bolt Mini Blot Modules (Life Technologies) for 60 minutes at 30V.

Blocking and detection of membranes was performed as previously described [49] with primary antibodies obtained from Agrisera (PsbA AS05-084 1:25,000; PsbD AS06-146 1:25,000; FtsH AS11-1789 1:5,000) and the secondary antibody, goat anti-rabbit HRP conjugated obtained from AbCam (Product number 6721). Blots were detected with ECL Select reagent (GE Healthscience) with a VersaDoc Imager (BioRad). Images were quantitated using Image Lab 4.0.1 software from BioRad. Four to five biological replicates of each strain/light/treatment combination were performed.

### Transcript analysis

#### Primer design

qRT-PCR primers were designed for each of the four isoforms of *ftsH* from *Prochlorococcus* MED 4, and *Prochlorococcus* MIT 9313 using sequences from CyanoBase, OligoNucleotide Calculator and Amplify 4X Software. Primers designed and used in this study are given in Table 1.

**Table 1:** Sequence-specific *ftsH* primers used for qRT-PCR, with experimentally determined optimal annealing temperature.

Strain	FtsH isoform	Forward/ Reverse	Annealing Tm (°C)	Primer sequence (5'-3')	Amplicon Size (bp)
MIT9313	FtsH 1	F	65	GCTCACAGGAGAGCAGCCGAGTAACG	178
MIT9313	FtsH 1	R	65	GCGATTGTCGAGTTCGGGATCTACGG	
MIT9313	FtsH 2	F	63	CCTGGCACGACTGAGCGGGAGAC	226
MIT9313	FtsH 2	R	63	CACCTGCCTGACGGGTTGGTTGAACAG	
MIT9313	FtsH 3	F	63	GCTTTTCAGCAGCTTCTTACCCAATCCTGC	208
MIT9313	FtsH 3	R	63	CAAGCGTTGGGGCAGATCCATATCGAAG	
MIT9313	FtsH 4	F	65	CCTTTGCGCCCTTCAAGCAGAAACCCAC	214
MIT9313	FtsH 4	R	65	TTCACCGTCAGAGAGGTATCAGCAGCC	
MED4	FtsH 1	F	63	TGATGGAGGTAGAAATGCTGTTATCGAAAC	157
MED4	FtsH 1	R	63	TTTCACAGGGTGAACGTCAAAACTTATTCC	
MED4	FtsH 2	F	63	AAGCTGTTCAAGATAAAGAAGTTAGCAGGG	157
MED4	FtsH 2	R	63	ACGGCTATATCTACATCATTCTCGGTTAGG	
MED4	FtsH 3	F	63	TTGGACTCGGAGCATTACTTTTATTCAGCA	173
MED4	FtsH 3	R	63	GGCTCCCTCTTCTGCTCCATTCAGTTCG	
MED4	FtsH 4	F	63	AGCGGCCAATTCGTTTGCATCAG	203
MED4	FtsH 4	R	63	CCTCTTTTAATTCTTCCGCGGCTTCAGG	

#### RNA isolation

Three 50 mL volumes of culture were spun down in sterile 50 mL Falcon tubes at 4200 rcf for 20 minutes in a JS 4.3 rotor (Beckman Coulter Avanti J-20 centrifuge) with 2.5–4 μL of 100X pluronic acid. The supernatant was removed until ~ 2 mL supernatant remained. Pellets were resuspended in remaining supernatant, pooled and transferred to a single nuclease free 2 mL microcentrifuge tube and centrifuged at 14,800 g for 5 minutes (Mikro 20 Hettich zentrifugen). The supernatant was removed, the pellet was flash frozen in liquid nitrogen and stored at -80°C for later use. The remaining volume of culture was shifted to 260 μmol photon m^-2^ s^-1^ (high light, HL) for 1.5 hours. The pelleting procedure was repeated for HL samples.

Cell pellets were resuspended in 1 mL Tri-reagent (Sigma-Aldrich) and left to incubate at room temperature for 5 minutes. One fifth the total volume of chloroform was added and shaken vigorously for 15 seconds. Samples were left to incubate at room temperature for 3 minutes, followed by centrifugation at 12,000 x g for 15 minutes at 4°C. Roughly 500 μL of the aqueous phase was removed carefully and transferred to a nuclease free 1.7 mL Eppendorf tube. A 500 μL volume of isopropanol was added and left to incubate at room temperature for 10 minutes, followed by centrifugation at 12,000 x g for 10 minutes at 4°C. The supernatant was removed leaving only the white RNA pellet. The RNA pellet was washed with 1 mL of 75% ethanol (prepared in nuclease free water) and vortexed briefly before centrifugation at 7500 x g for 5 minutes at 4°C. The wash was discarded and the pellet was left to air dry for 15 minutes. The pellet was resuspended in nuclease free water, and incubated at room temperature for 15 minutes. RNA was treated with TURBO DNA-free kit, a DNase enzyme to remove unwanted genomic DNA, per the ThermoFisher Scientific protocol. RNA samples were stored at -80°C until further use. Samples were run on an Agilent Bioanalyzer to assess RNA quality and integrity.

High quality RNA extracts were obtained for both *Prochlorococcus* strains, however, despite attempting a number of different isolation procedures, we were unable to obtain RNA from *Synechococcus* WH 8102 that was of high enough quality for qRT-PCR.

#### First strand synthesis

Complementary DNA (cDNA) was synthesized using the BioRad iScript cDNA Synthesis Kit. Equal amounts of RNA (1000 ng for MIT 9313 and 1600 ng for MED 4) were reverse transcribed in RT+ and RT- samples based on the biological sample with the lowest concentration of RNA. Final ng RNA/μL in cDNA synthesis reaction was equivalent for RT+ and RT-. First strand synthesis protocol was as follows: 5 minutes at 25°C, 20 minutes at 46°C, 1 minute at 95°C, hold at 4°C.

#### Quantitative Real Time Reverse Transcriptase Polymerase Chain Reaction (qRT-PCR)

qRT-PCR was performed using the Bioline SensiFast SYBR No-ROX 2X master mix, BioRad CFX96 Touch real-time PCR detection system and software. The template concentration was the same for all reactions (0.6 ng RNA/μL). One *ftsH* isoform of one picocyanobacterial strain of all biological samples was run on a 96 well plate at a time. Triplicate assays of each *ftsH* isoform for Low growth light (LL) RT+, LL RT-, after 90 minutes of high light (260 μmol photons m^-2^ s^-1^) (HL) RT+ and HL RT-were performed for 6 biological replicates. The CFX96 manager software generated transcriptional analysis data using the ΔC_q_ method and a melt curve was added to the end of each run. The products of qRT-PCR were confirmed by agarose gel electrophoresis of the reactions with staining of the DNA by SYBR Safe DNA Stain (BioRad).

### Bioinformatic analysis

Amino acid sequences of FtsH 1, 2, 3 and 4 from the three picocyanobacterial species and the cyanobacterium *Synechocystis sp*. *PCC6803* were obtained as FASTA files from CyanoBase, http://genome.microbedb.jp/cyanobase/ [50]. Multiple alignment was performed using both ClustalW [51] and MUSCLE [52] algorithms, implemented in the MEGA software package (version 7.0.26)[53]. For each alignment method, the best-fit substitution models were determined using a maximum likelihood method in MEGA, with a 95% site coverage cut-off treatment for missing data.

Phylogenetic trees were generated from both ClustalW and MUSCLE alignments using a variety of statistical methods in the MEGA software package. A maximum parsimony method was used with 1000 bootstrap replicates, and a 95% site coverage cut-off treatment for missing data. Maximum likelihood trees were created using several of the previously determined best-fit substitution models, including LG [54] and WAG [55], all with 1000 bootstrap replicates and a 95% site coverage cut-off. Neighbour-joining trees were created using the Jones-Taylor-Thornton substitution model [56], with gamma distributed evolutionary substitution rates among sites, 1000 bootstrap replicates, and a 95% site coverage cut off. When creating the neighbour-joining trees, a maximum likelihood method was first used to estimate the α parameter for gamma distributed substitution rates.

### Data handling, plotting and statistical analysis

For an estimate of _kPsbA_, the rate constant for removal of PsbA protein, the following equation was used: {ln[PsbA]_T = 0_ –ln[PsbA]_T linco_/T treatment}, where T = 0 is the sample before treatment, T linco is the sample treated with lincomycin, and T treatment is the time under exposure to treatment (in seconds). Similarly, k_PSIIactive_, the rate constant for the loss of PSII Active, was calculated using the equation: {ln[PSIIactive]_T = 0_ –ln[PSIIactive]_T linco_/T treatment}.

Data were imported into R Studio [40] for statistical analysis and generation of figures with the ggplot2 package [57].

## Results & discussion

### Cross calibration of quantitative immunoblotting and activity measurements of PSII

**Fig 1:**
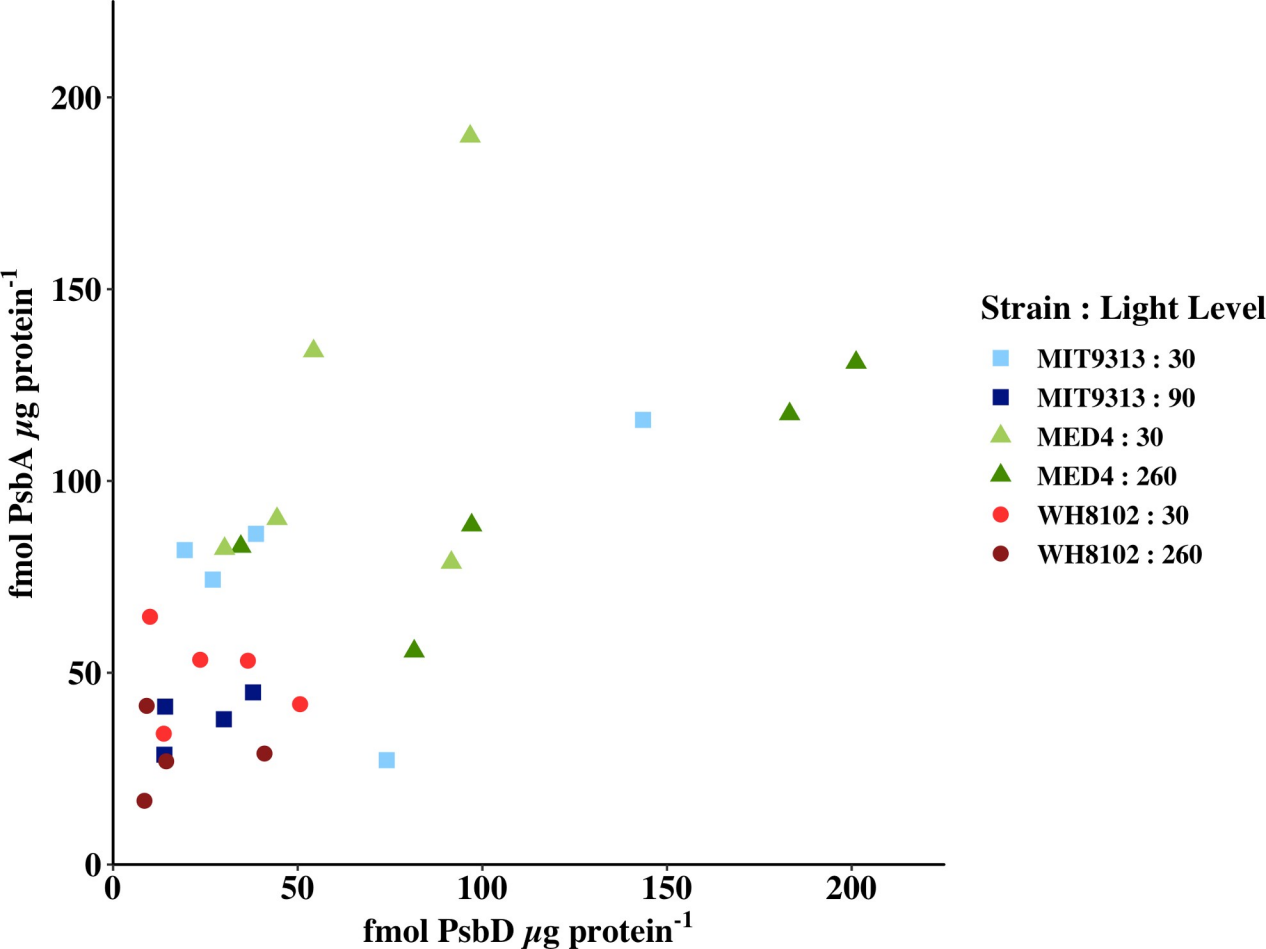
PsbA plotted versus PsbD, both in fmoles μg protein^-^1 as determined by quantitative immunoblotting. MIT 9313 grown at 30 μmol photons m^-2^ s^-1^ (pale blue squares); MIT 9313 grown at 90 μmol photons m^-2^ s^-1^ (dark blue squares); MED 4 grown at 30 μmol photons m^-2^ s^-1^ (pale green triangles); MED 4 grown at 260 μmol photons m^-2^ s^-1^ (dark green triangles); WH 8102 grown at 30 μmol photons m^-2^ s^-1^ (light red circles); WH 8102 grown at 260 μmol photons m^-2^ s^-1^ (dark red circles).

This study was designed to link changes in physiological measurements of PSII activity with the changes in the protein components and the enzyme system responsible for PsbA turnover. PSII is made up of a large number of subunits, but one PsbA protein (sometimes referred to as D1) and one PsbD protein (sometimes referred to as D2) make up the core of the reaction centre. These proteins are present stoichiometrically in a 1 PsbA:1 PsbD ratio in the monomeric PSII complex. As the absolute quantitation of PSII by immunoblotting relies on different antibodies and calibrated standards for the two proteins, we first compared the amounts of PsbA and PsbD in the three strains of picocyanobacteria grown at two different light levels each. The results are presented in Fig 1 which plots PsbA in fmoles μg protein^-1^ versus PsbD in fmoles μg protein^-1^ for samples at time zero before any high light treatment. For fully functional PSII a ratio of 1:1 is expected. The independently calibrated standards generate similar estimates of number of PSII complexes in the samples (linear regression line gives a slope of 0.5 and an intercept of 42, R^2^ = 0.398). The y-intercept of 42 fmoles PsbA μg protein^-1^ in the absence of PsbD, suggests a surplus of PsbA over PsbD on average. The average ratio of PsbA:PsbD ± SEM at the growth light is 2.1 ± 0.7 for MIT 9313 grown at 30 μmol photons m^-2^ s^-1^; 1.9 ± 0.4 for MIT 9313 grown at 90 μmol photons m^-2^ s^-1^; 2.0 ± 0.3 for MED 4 grown at 30 μmol photons m^-2^ s^-1^; 1.1 ± 0.3 for MED 4 grown at 260 μmol photons m^-2^ s^-1^; 3.2 ± 1.1 for WH 8102 grown at 30 μmol photons m^-2^ s^-1^; and 2.3 ± 0.8 for WH 8102 grown at 260 μmol photons m^-2^ s^-1^. These ratios indicate that in all cases, on average there is more PsbA than PsbD. Models of PSII repair include removal of damaged PsbA and synthesis of new PsbA into PSII to replace damaged PsbA [30]. If the clearance of PsbA does not match the rate of synthesis, accumulation of excess PsbA is expected. It should be noted that the lower PsbA and PsbD content expressed per μg protein in *Synechococcus* likely reflects a denominator effect given the abundance of the protein-rich light harvesting phycobilisomes in this species, and the absence of phycobilisomes in *Prochlorococcus*.

**Fig 2:**
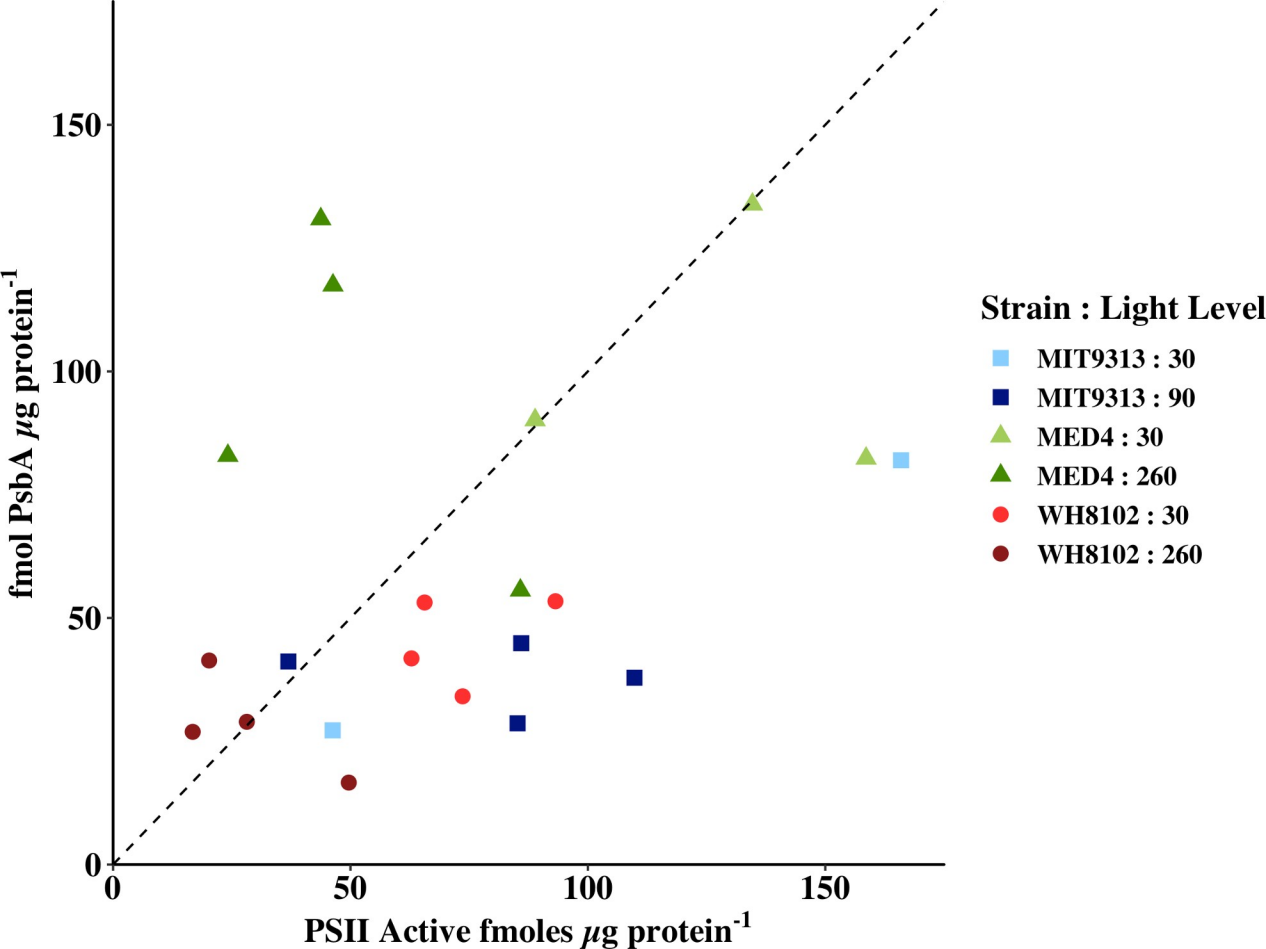
PsbA in fmoles μg protein^-1^ is plotted versus PSII Active in fmoles μg protein^-1^. MIT 9313 grown at 30 μmol photons m^-2^ s^-1^ (pale blue squares); MIT 9313 grown at 90 μmol photons m^-2^ s^-1^ (dark blue squares); MED 4 grown at 30 μmol photons m^-2^ s^-1^ (pale green triangles); MED 4 grown at 260 μmol photons m^-2^ s^-1^ (dark green triangles); WH 8102 grown at 30 μmol photons m^-2^ s^-1^ (light red circles); WH 8102 grown at 260 μmol photons m^-2^ s^-1^ (dark red circles). A 1:1 line is included.

Oxygen flash yield measurements [28] were made on the samples to determine the number of active PSII reaction centres ([PSII_active_]). When expressed per μg protein, we can see that the two completely independent measures of PSII give results in the same order of magnitude while not clustering tightly around the 1:1 line (Fig 2, for samples at time zero, without high light or lincomycin treatment). Three samples of MIT 9313 grown at 30 μmol photons m^-2^ s^-1^ where the number of PSII active measured impossibly widely exceeded the number of reaction centre proteins measured were removed from the analysis. As the number of fmoles of PSII active centres per μg protein is calculated from the concentration of centres per mL (μmoles PSII/mL culture * mL culture pelleted *10^9^ fmoles/μmole ÷ μL extract volume ÷ μg protein μL^-1^), it is highly dependent on the measured protein concentration of the extract. The three outlying points for MIT 9313 all had very low measured protein concentrations, but typical [PSII_active_]. The measurements of protein concentration were repeated numerous times, thus it seems likely that the pellets from these samples were incompletely disrupted, yielding less total protein than was actually present.

### FtsH Hexamers: PsbA differ between strains and at different growth lights

**Fig 3:**
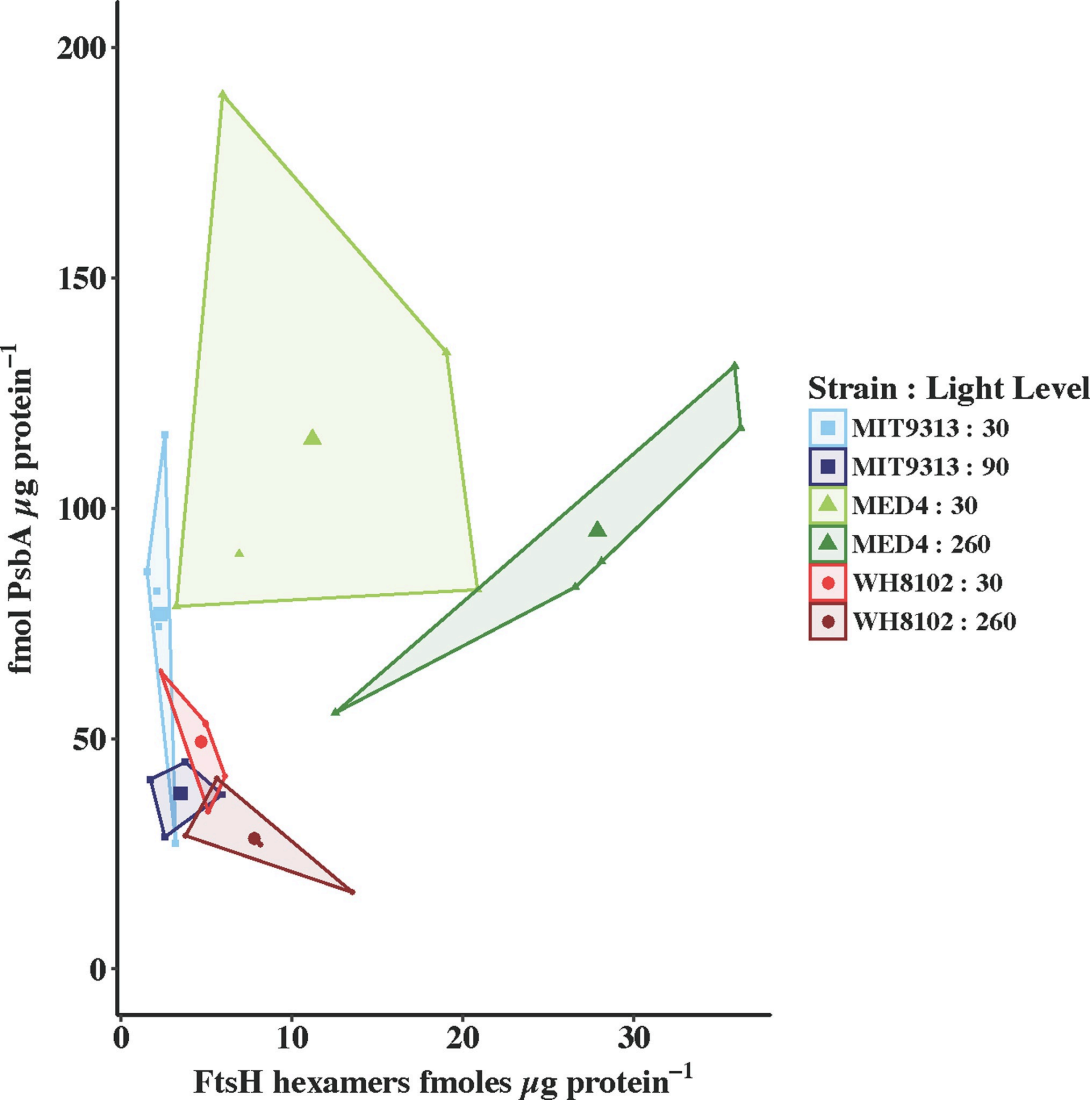
PsbA plotted versus total FtsH hexamers, both in fmoles μg protein^-1^. MIT 9313 grown at 30 μmol photons m^-2^ s^-1^ (pale blue squares); MIT 9313 grown at 90 μmol photons m^-2^ s^-1^ (dark blue squares); MED 4 grown at 30 μmol photons m^-2^ s^-1^ (pale green triangles); MED 4 grown at 260 μmol photons m^-2^ s^-1^ (dark green triangles); WH 8102 grown at 30 μmol photons m^-2^ s^-1^ (light red circles); WH 8102 grown at 260 μmol photons m^-2^ s^-1^ (dark red circles). The data are presented as a convex hull (boundary box of outermost points in the group) of groups with the mean value added as a larger symbol to the centre.

Using a global antibody that evenly detects all FtsH isoforms in all picocyanobacterial strains, we can measure the total number of FtsH monomers, and then infer the hexamer content in the samples. In Fig 3 we plot PsbA in fmoles μg protein^-1^ versus the number of total FtsH hexamers in fmoles μg protein^-1^ for samples at time zero (no high light treatment or lincomycin treatment). MIT 9313 grown at low light has the lowest amount of detectable FtsH protein, with an average FtsH hexamer to PsbA ratio 0.030 (or 33 PSII served by one FtsH hexamer). When grown at 90 μmol photons m^-2^ s^-1^, MIT 9313 has less PsbA and ~50% more FtsH, giving an average FtsH hexamer to PsbA ratio of 0.092 (or 11 PSII served by one FtsH hexamer). MED 4 on the other hand has more FtsH hexamers than MIT 9313 with a ratio of FtsH hexamers:PsbA of 0.097 (or 10 PSII served by one FtsH hexamer) at 30 μmol photons m^-2^ s^-1^ and 0.293 (or just over 3 PSII served by one FtsH hexamer) at 260 μmol photons m^-2^ s^-1^. The increased ratio of FtsH hexamers to PsbA in MED 4 grown in high light is almost entirely due to a higher level of FtsH rather than a difference in PsbA content at the higher growth light. WH 8102 achieves FtsH hexamer:PsbA ratios similar to MED 4 (0.095 at 30 μmol photons m^-2^ s^-1^ and 0.273 at 260 μmol photons m^-2^ s^-1^) however the change in ratio is brought about by a balanced decrease in PsbA with an increase in FtsH rather than largely through an increase in FtsH. For the purpose of comparison, we measured the ratio of FtsH hexamers to PsbA in the freshwater model organism *Synechocystis* PCC 6803 grown at 30 μmol photons m^-2^ s^-1^ and determined that this strain has a ratio of 0.15 (~ 7 PSII served by one FtsH hexamer). One has to bear in mind that this strain grows much more quickly than the picocyanobacterial strains and at a higher temperature.

### PsbD is removed at rates similar to PsbA

**Fig 4:**
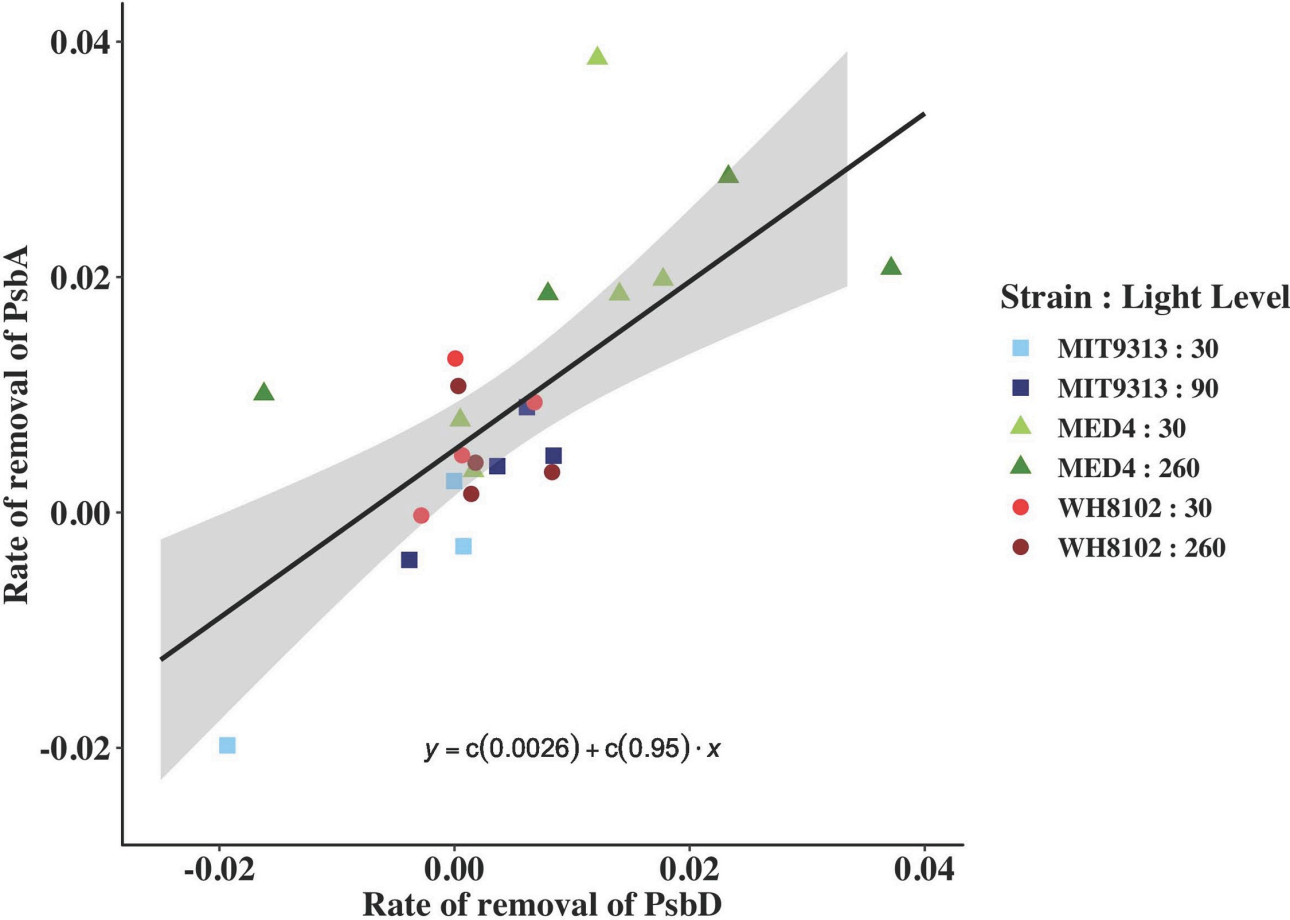
Rate of removal of PsbA in fmoles μg protein^-1^ s^-1^ is plotted versus the rate of removal of PsbD in fmoles μg protein^-1^ s^-1^. MIT9313 grown at 30 μmol photons m^-2^ s^-1^ (pale blue squares); MIT9313 grown at 90 μmol photons m^-2^ s^-1^ (dark blue squares); MED4 grown at 30 μmol photons m^-2^ s^-1^ (pale green triangles); MED4 grown at 260 μmol photons m^-2^ s^-1^ (dark green triangles); WH8102 grown at 30 μmol photons m^-2^ s^-1^ (light red circles); WH8102 grown at 260 μmol photons m^-2^ s^-1^ (dark red circles). A line regression line with 95% confidence intervals is included.

We calculated the rate of removal of the two core proteins of the PSII reaction centre at high light (260 μmol photons m^-2^ s^-1^) as the product of the rate constant for their loss in the presence of a lincomycin treatment (for calculation, see Materials & Methods) and the content of the protein relative to total cellular protein. Fig 4 shows the rate of removal of PsbA in fmoles μg protein^-1^s^-1^ versus the rate of removal of PsbD in fmoles μg protein^-1^s^-1^. The slope of the linear regression falling through these points is 0.95, indicating that the rates of removal are nearly identical for all of the strains and growth light combinations, challenging the oft held notion that PsbA is preferentially removed from the PSII reaction centre over PsbD, but similar to findings in diatoms [58,59].

### MIT 9313 suffers rapid loss of active PSII centres and slow PsbA removal

**Fig 5:**
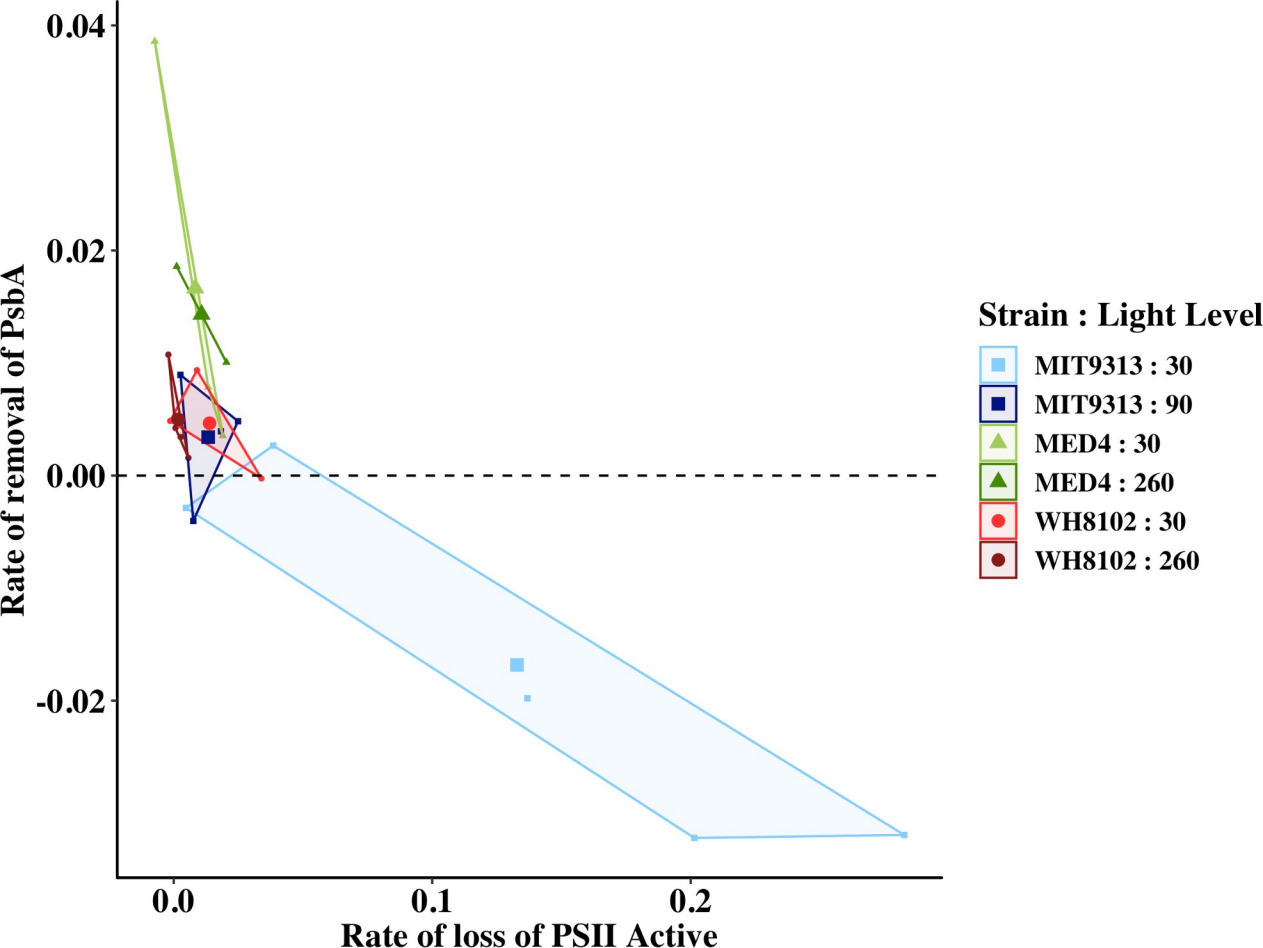
Rate of removal of PsbA versus the rate of loss of active PSII both in fmoles μg protein^-1^ s^-1^. MIT 9313 grown at 30 mol photons m^-2^ s^-1^ (pale blue squares); MIT 9313 grown at 90 μmol photons m^-2^ s^-1^ (dark blue squares); MED 4 grown at 30 μmol photons m^-2^ s^-1^ (pale green triangles); MED 4 grown at 260 μmol photons m^-2^ s^-1^ (dark green triangles); WH 8102 grown at 30 μmol photons m^-2^ s^-1^ (light red circles); WH 8102 grown at 260 μmol photons m^-2^ s^-1^ (dark red circles). The data are presented as a convex hull (boundary box of outermost points in the group) of groups with the mean value added as a larger symbol to the centre.

We can calculate the rate of loss of Active PSII in the presence of high light and lincomycin similarly to the way we calculate the rate of removal of PsbA, as the product of the rate constant for PSII loss and the concentration of active PSII at the beginning of the high light treatment. Fig 5 shows that in MIT 9313 the average rate of PSII loss is faster than the average rate of PsbA removal at both growth lights, but particularly when grown at 30 μmol photons m^-2^ s^-1^, where very rapid loss of active PSII centres occurs with an immeasurably slow rate of PsbA removal. The average rate of loss of PsbA in fmoles μg protein^-1^ s^-1^ ± SEM when shifted to high light in the presence of lincomycin is -1.7 x 10^−2^ ± 7.2 x 10^−3^ for MIT 9313 grown at 30 μmol photons m^-2^ s^-1^; 3.4 x 10^−3^ ± 2.7 x 10^−3^ for MIT 9313 grown at 90 μmol photons m^-2^ s^-1^; 1.8 x 10^−2^ ± 6.1 x 10^−3^ for MED 4 grown at 30 μmol photons m^-2^ s^-1^; 1.7 x 10^−2^ ± 4.0 x 10^−3^ for MED 4 grown at 260 μmol photons m^-2^ s^-1^; 6.8 x 10^−3^ ± 2.9 x 10^−3^ for WH 8102 grown at 30 μmol photons m^-2^ s^-1^; and 5.0 x 10^−3^ ± 2.9 x 10^−3^ for WH 8102 grown at 260 μmol photons m^-2^ s^-1^ (Table 2 below).

**Table 2:** Rates of PsbA and PsbD removal and PsbA and PsbD synthesis.

Strain	Growth Light	Rate of PsbA removal	Rate of PsbA synthesis	Rate of PsbD removal	Rate of PsbD synthesis
MIT 9313	30	**-1.7 x 10**^**−2**^ ± *7*.*2 x 10*^*−3*^	**-1.8 x 10**^**−2**^ ± *1*.*2 x 10*^*−2*^	**-1.0 x 10**^**−2**^ ± *4*.*8 x 10*^*−3*^	**-1.9 x 10**^**−2**^ ± *1*.*0 x 10*^*−2*^
MIT 9313	90	3.4 x 10^−3^ ± *2*.*7 x 10*^*−3*^	3.2 x 10^−3^ ± *1*.*9 x 10*^*−3*^	3.6 x 10^−3^ ± *2*.*7 x 10*^*−3*^	1.9 x 10^−3^ ± *9*.*0 x 10*^*−4*^
MED 4	30	1.8 x 10^−2^ ± *6*.*1 x 10*^*−3*^	2.8 x 10^−3^ ± *4*.*6 x 10*^*−3*^	9.2 x 10^−3^ ± *3*.*1 x 10*^*−3*^	9.5 x 10^−3^ ± *8*.*7 x 10*^*−3*^
MED 4	260	1.7 x 10^−2^ ± *4*.*0 x 10*^*−3*^	1.3 x 10^−2^ ± *2*.*7 x 10*^*−3*^	1.1 x 10^−2^ ± *9*.*1 x 10*^*−3*^	6.2 x 10^−3^ ± *7*.*3 x 10*^*−3*^
WH 8102	30	6.8 x 10^−3^ ± *2*.*9 x 10*^*−3*^	2.4 x 10^−3^ ± *9*.*6 x 10*^*−4*^	1.2 x 10^−3^ ± *2*.*0 x 10*^*−3*^	4.9 x 10^−4^ ± *1*.*1 x 10*^*−3*^
WH 8102	260	5.0 x 10^−3^ ± *2*.*0 x 10*^*−3*^	3.8 x 10^−3^ ± *1*.*3 x 10*^*−3*^	3.0 x 10^−3^ ± *1*.*8 x 10*^*−3*^	2.6 x 10^−3^ ± *1*.*3 x 10*^*−3*^

All in fmoles ug protein^-1^s-1 ± S.E.M.

### Picocyanobacteria increase oxygen evolution per PSII when shifted to high light

To determine how MIT 9313 may be coping with the rapid loss of PSII active centres, we calculated the rate of oxygen evolution per PSII per second at the beginning of the high light treatment and again at the end, after 49 minutes at 260 μmol photons m^-2^ s^-1^. All strains grown at 30 μmol photons m^-2^ s^-1^ increase their oxygen evolution per PSII after being shifted to the higher light level (S1 Table). No such effect was seen for cells grown at high light. The effect is particularly significant for MIT 9313 grown at 30 μmol photons m^-2^ s^-1^, which doubled its oxygen evolution per remaining PSII per second after it has been exposed to high light for 49 minutes. The effect appears to be similar to the one presented by Behrenfeld et al [60] who showed increases in electron turnover (1/τ_PSII_) when the number of PSII centres decreased as a result of photoinhibition. In that study, cells grown at low light had initial electron turnover rates that were lower than for cells grown under high light, but which could increase upon loss of PSII centres [60].

Table 3 presents the rate constant data for photoinactivation (k_PI_, determined by fluorescence measurements), repair (k_REC_, determined by fluorescence measurements), PSII loss (k_PSII_, determined by oxygen flash yield) and PsbA removal (k_PsbA_, determined by quantitative immunoblotting) all in s^-1^. All strains except for WH 8102 grown at 260 μmol photons m^-2^ s^-1^ show similar rate constants for photoinactivation of 1.6–2.2 x 10^−4^ s^-1^, consistent with those reported by Soitamo et al [29]. WH 8102 grown at 260 μmol photons m^-2^ s^-1^ has a rate constant approximately half of that, consistent with the presence of photoprotective effects in cells grown at this light level [28]. The rate constants for the repair of PSII differ between strains. MIT 9313 grown at 30 μmol photons m^-2^ s^-1^ shows no measurable rate constant for repair (-k_REC_), although it does show measurable repair when grown at 90 μmol photons m^-2^ s^-1^, indicating that the repair machinery can be induced when the organism grows at higher light. The k_REC_ values reported herein are slightly higher than those reported by Six et al for picocyanobacteria [27] but they show the same trends with WH 8102 having a faster k_REC_ than MED 4/PCC 9511, and these are in turn much faster than MIT 9313 or SS120. The rate constants for the loss of functional PSII were similar for all strains except for WH 8102 grown at 260 μmol photons m^-2^ s^-1^, ranging from 1.9–3.0 x 10^−4^ s^-1^. As for the rate constant for photoinactivation, WH 8102 grown at 260 μmol photons m^-2^ s^-1^ shows a rate constant for loss of PSII roughly one order of magnitude slower than for the other strain/light level combinations indicating the presence of a photoprotective strategy such as the induction of a high light tolerant PsbA isoform [61]. MED 4 and WH 8102 have similar rate constants for the removal of PsbA of 1.2–1.7 x 10^−4^ s^-1^. MIT 9313 has no measurable rate constant for PsbA removal when grown at 30 μmol photons m^-2^ s^-1^, however, it is able to achieve a modest PsbA removal when grown at 90 μmol photons m^-2^ s^-1^. Mella-Flores et al [62] reported similar differences in repair rates between *Synechococcus* WH 7803 and *Prochlorococcus* PCC 9511 (similar to MED 4) under variable light as we report here between WH 8102 and MED 4 [62]. In addition to differences in PsbA and PsbD pool sizes, quantum yields for PSII and repair rates, Mella-Flores also [62] report that the induction of *ftsH* transcripts in *Synechococcus* is much greater than that of *Prochlorococcus* when shifted into the light from the dark, and that this induction is stronger in the presence of UV [62]. In *Prochlorococcus* PCC 9511 they showed less induction of *ftsH* when shifted to the light in the presence of UV and this correlated with a slower rate of PSII repair.

**Table 3:** Rate constants for PSII photoinactivation (k_PI_), repair (k_REC_), PSII loss (k_PSII_) and PsbA removal (k_PsbA_).

Strain	Growth Light	K_PI_ (s^-1^) fluorescence	K_REC_ (s^-1^) fluorescence	k_PSII_ (s^-1^) oxygen flash yield	k_PsbA_ (s^-1^) quantitative immunoblotting
MIT 9313	30	1.6 x 10^−4^ *± 1*.*3 x 10*^*−5*^	**-3.6 x 10**^**−5**^ *± 5*.*0 x 10*^*−5*^	3.0 x 10^−4^ *± 7*.*8 x 10*^*−5*^	**-2.0 x 10**^**−4**^ *± 7*.*2 x 10*^*−5*^
MIT 9313	90	2.2 x 10^−4^ *± 7*.*6 x 10*^*−6*^	2.7 x 10^−4^ *± 5*.*6 x 10*^*−5*^	1.9 x 10^−4^ *± 4*.*0 x 10*^*−5*^	7.2 x 10^−5^ *± 7*.*6 x 10*^*−5*^
MED 4	30	1.7 x 10^−4^ *± 9*.*2 x 10*^*−6*^	2.3 x 10^−4^ *± 3*.*2 x 10*^*−5*^	2.2 x 10^−4^ *± 1*.*5 x 10*^*−4*^	1.5 x 10^−4^ *± 4*.*9 x 10*^*−5*^
MED 4	260	1.2 x 10^−4^ *± 9*.*9 x 10*^*−6*^	1.1 x 10^−3^ *± 8*.*9 x 10*^*−5*^	1.9 x 10^−4^ *± 1*.*3 x 10*^*−4*^	1.7 x 10^−4^ *± 3*.*2 x 10*^*−5*^
WH 8102	30	2.1 x 10^−4^ *± 1*.*7 x 10*^*−5*^	8.9 x 10^−4^ *± 1*.*6 x 10*^*−4*^	2.6 x 10^−4^ *± 1*.*3 x 10*^*−4*^	1.2 x 10^−4^ *± 4*.*7 x 10*^*−5*^
WH 8102	260	7.8 x 10^−5^ *± 1*.*2 x 10*^*−5*^	7.1 x 10^−4^ *± 1*.*1 x 10*^*−4*^	3.7 x 10^−5^ *± 4*.*9 x 10*^*−5*^	1.6 x 10^−4^ *± 3*.*6 x 10*^*−5*^

All in s^-1^ ± S.E.M.

**Fig 6:**
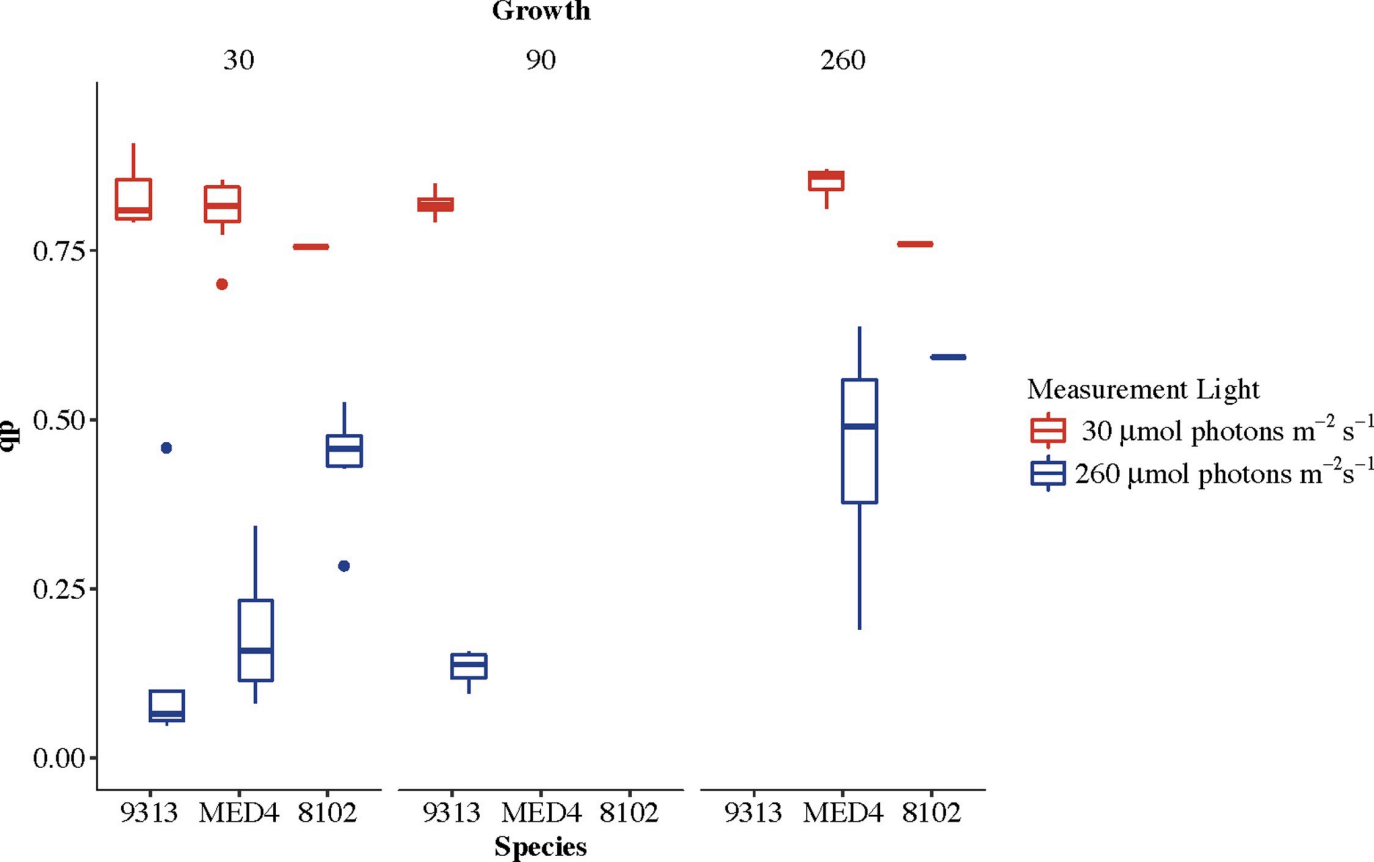
The fraction of PSII centres that are open (qp) is plotted at 30 μmol photons m^-2^ s^-1^ (red) and then in the first step of the treatment light (260 μmol photons m^-2^ s^-1^) (blue).

As Murphy et al 2017 have shown a positive correlation between excitation pressure (1-qp) and the target size for photoinactivation and the yield of PSII photoinactivations per photon delivered to PSII for photochemistry [28], we calculated the fraction of open PSII centres immediately before and after the shift from 30 μmol photons m^-2^ s^-1^ to 260 μmol photons m^-2^ s^-1.^ Fig 6 shows that both *Prochlorococcus* strains grown at 30 μmol photons m^-2^ s^-1^ show a large and very rapid drop in qp when shifted to higher light. This effect is much less pronounced in *Synechococcus*. Even when grown at 90 μmol photons m^-2^ s^-1^ MIT 9313 shows a large closure of PSII upon the shift to higher light which may explain this strain's heightened sensitivity to photoinactivation. MED4 and WH 8102 grown at 260 μmol photons m^-2^ s^-1^ do not show a large drop in qp when shifted from 30 to 260 μmol photons m^-2^ s^-1^, reflecting successful acclimation to higher growth light.

Table 2 presents the mean rates of PsbA and PsbD removal and the rates of PsbA and PsbD synthesis (calculated as (PsbA or PsbD after treatment in the absence of lincomycin–PsbA or PsbD after treatment in the presence of lincomycin) ÷ time of treatment in s). While MIT 9313 grown at 30 μmol photons m^-2^ s^-1^ is unable to remove damaged PsbA or PsbD, when grown at 90 μmol photons m^-2^ s^-1^ it has well matched PsbA and PsbD removal and synthesis rates. Of all of the strains, MED 4 shows the fastest rates of removal and synthesis of the PSII proteins. One should bear in mind though that these rates depend on the concentration of the proteins on a per μg total protein basis, and MED 4 has the highest PSII protein content per total protein, given the lack of phycobilisomes proteins in the whole cell protein pool. Overall, with the exception of low light grown MIT 9313, the strains have well matched rates of PsbA and PsbD removal and synthesis. Cells grown under the lower light appear to lag in the rate of synthesis, but this is more balanced when cells are grown at high light.

That the calculated rate of PsbA and PsbD accumulation in MIT 9313 grown at 30 μmol photons m^-2^ s^-1^ is negative, suggests that when these cells are shifted to the higher light in the presence of lincomycin, they are so deficient in FtsH (capacity to remove PsbA) that they actually accumulate more PsbA than in the absence of lincomycin. The undetectably low rate of PsbA and PsbD removal in low light grown MIT 9313 allows us a different method to calculate the rate of accumulation of PsbA and PsbD in the absence of lincomycin (final PsbA/PsbD–initial)/time of treatment). We determine that the rate of PsbA accumulation is 9.6 x 10^−3^ fmoles μg protein^-1^ s^-1^ and that for PsbD is 8.6 x 10^−3^ fmoles μg protein^-1^ s^-1^. These values are similar to those shown in Table 2 for MED 4 grown at 30 μmol photons m^-2^ s^-1^.

Taken together, the results of Figs 3 and 5 and Tables 2 and 3 indicate that low light grown MIT 9313 is unable to perform PSII repair at measurable rates likely due the lack of the enzymatic machinery to remove damaged PsbA from that reaction centre. The enzymatic machinery normally thought to be responsible for the removal of damaged PsbA from PSII is an FtsH heterohexamer. From Fig 3 it is clear that the level of FtsH relative to PsbA is very low in this strain grown at this light level.

### MIT 9313 and MED 4 differ in their FtsH isoform expression levels

**Fig 7:**
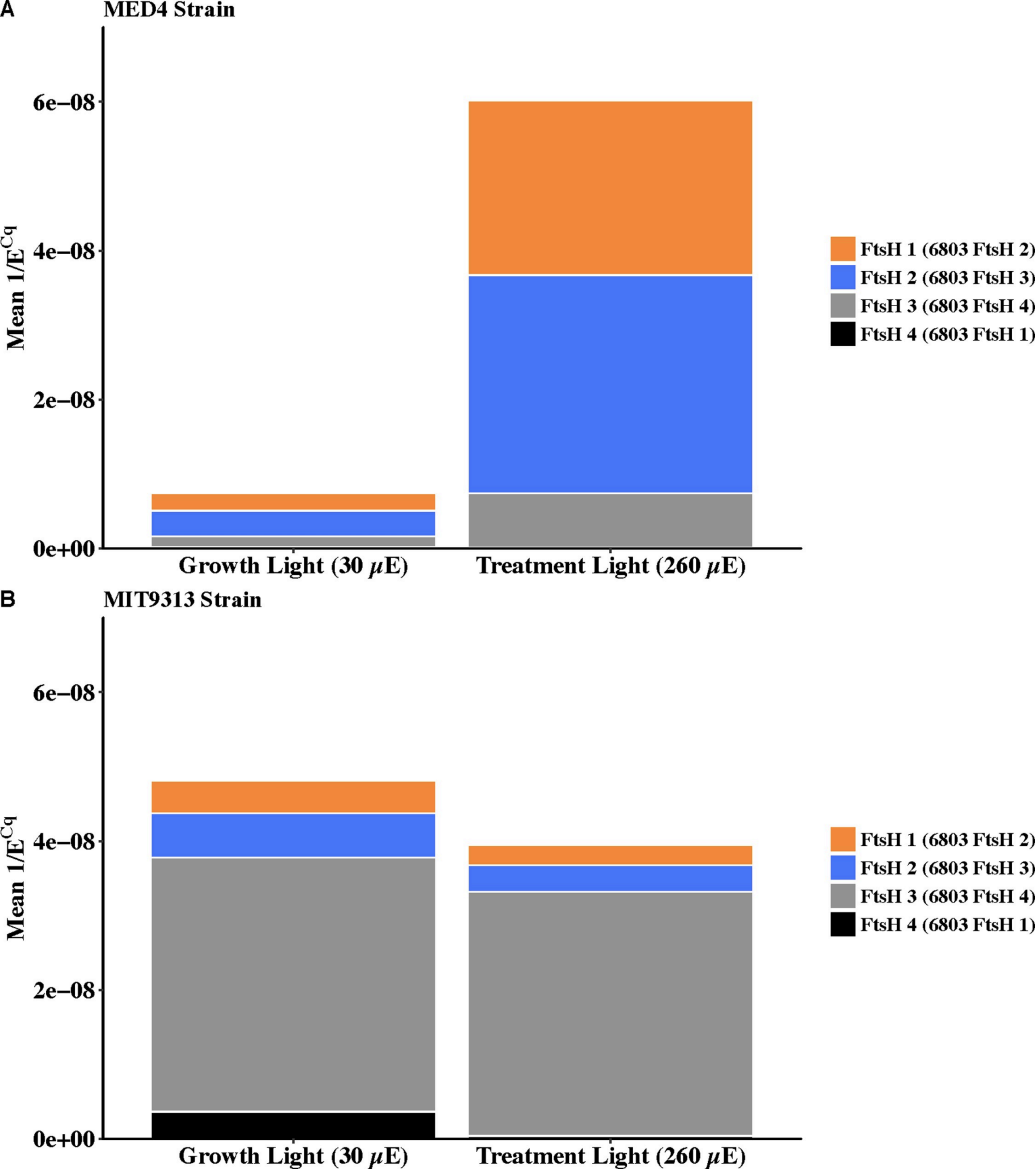
Stacked bar graph showing the relative number of transcripts in the FtsH pool as determined by 1/E^Cq^. Cells were grown at 30 μmol photons m^-2^ s^-1^ (Growth Light) and shifted to the Treatment Light of 260 μmol photons m^-2^ s^-1^ for 90 minutes. Values presented are the mean of means of 5 biological replicates each performed with three technical replicates.

To explore differences in the ability of the strains to alter their expression of FtsH isoforms when subject to higher light conditions, we performed Quantitative Real Time Reverse Transcriptase Polymerase Chain Reaction (qRT-PCR) on strains grown at 30 μmol photons m^-2^ s^-1^ and shifted to 260 μmol photons m^-2^ s^-1^ for 90 minutes. Unfortunately, we were unable to isolate RNA of high enough quality for qRT-PCR for WH 8102 despite attempting a number of isolation protocols. The results from the two *Prochlorococcus* strains, however, do show marked differences in the isoforms expressed and the induction by a short high light treatment. Fig 7 shows a stacked bar chart with the relative number of transcripts of each isoform depicted at both the growth light of 30 μmol photons m^-2^ s^-1^ and upon a shift to 260 μmol photons m^-2^ s^-1^ for 90 minutes. As 1/E^Cq^ is relative to the number of transcripts in the pool, we can infer that at the low growth light, MIT 9313 has a much larger FtsH transcript pool, but it is predominantly the annotated FtsH 3 isoform, while MED 4 has a much smaller total FtsH transcript pool that is more evenly balanced between the annotated 1, 2 and 3 isoforms. Following the high light treatment, the FtsH transcript pool has not increased in MIT 9313 (has possibly decreased slightly) while the MED 4 transcript pool increased 8-fold, mostly the annotated FtsH 1 and 2 isoforms.

**Fig 8:**
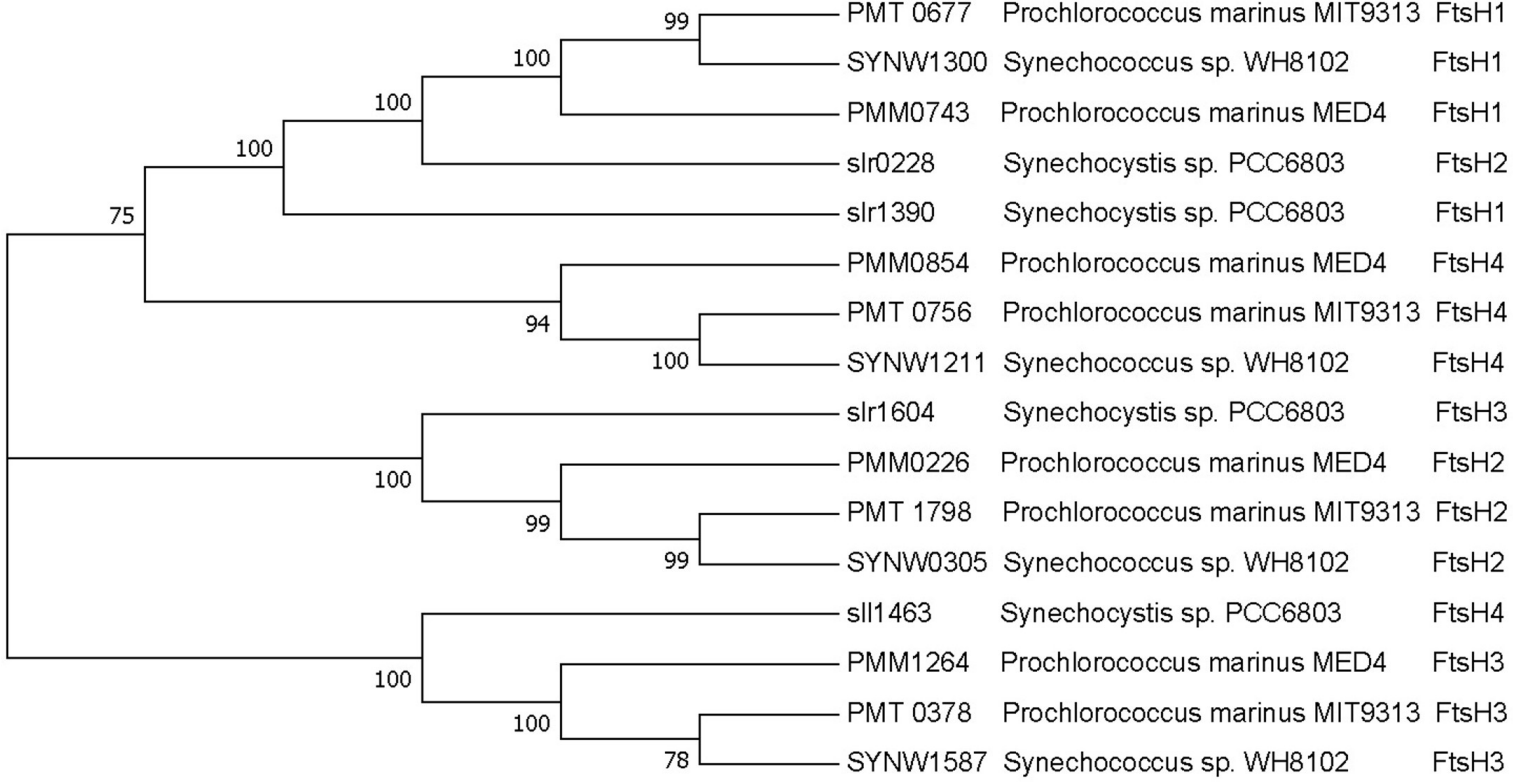
Phylogenetic tree generated using MUSCLE with the maximum likelihood method. See Materials and Methods for description.

In order to understand the significance of these differences, we performed a bioinformatic comparison of the annotated FtsH isoforms in the picocyanobacteria relative to those of the model freshwater cyanobacterium *Synechocystis* PCC 6803. A number of trees were generated using clustalW and MUSCLE, but all showed results similar to that depicted in Fig 8 which shows that picocyanobacterial FtsH 1 is homologous to PCC 6803 FtsH 2; picocyanobacterial FtsH 2 is homologous to PCC 6803 FtsH 3; picocyanobacterial FtsH 3 is homologous to PCC 6803 FtsH 4; and picocyanobacterial FtsH 4 was homologous to PCC 6803 FtsH 1, although this relationship was less obvious than the others.

As it is a heterohexamer of FtsH 2 and 3 from *Synechocystis* 6803 that is thought to be responsible for removal of damaged PsbA [30] it is not surprising that MED 4, with its rapid rate of PSII repair and PsbA removal, has a considerable amount of total FtsH protein and that this strain expresses the isoforms homologous to *Synechocystis* FtsH 2 and 3. The lack of expression of isoforms homologous to *Synechocystis* FtsH 2 and 3 in MIT 9313, with expression predominantly of the *Synechocystis* FtsH 4 type, suggests that the small FtsH protein pool in MIT 9313 grown at low light is predominantly a homohexamer of the homolog to *Synechocystis* FtsH 4 rather than a heterohexamer of homologs to the *Synechocystis* FtsH 2 and FtsH 3 mediating PSII repair. As MIT 9313 has no measurable rate of PsbA removal or PSII repair when grown at 30 μmol photons m^-2^ s^-1^, it is likely that the low FtsH protein levels and the different *ftsH* isoform expression are responsible for this difference. The roles of the *Synechocystis* FtsH 4 isoform in the PSII repair process, if any, have not been elucidated. A heterohexamer of FtsH 1 and 3 has been shown to regulate the response of *Synechocystis* to iron limitation by controlling the levels of transcriptional regulators [63]. FtsH 4 has been shown to form a homohexamer in *Synechocystis* [33], but the functional role of this isoform is not clear and has been shown to be non-essential for survival through gene knock out experiments [64]. In *Synechocystis*, FtsH 4 homohexamers are present in thylakoid membranes [65] with most of the localization at the peripheral region of the thylakoid membranes [66]. Interestingly, when *Synechocystis* is shifted to high light, the FtsH 3 and 4 isoforms are observed to shift their localization inwards from the periphery of the thylakoid membrane [66]. This localization of FtsH 4 does suggest a role for this isoform in photosynthesis and in responses to the high light challenge. Given the lack of PSII repair capacity for MIT 9313 grown at low light, which has little FtsH and likely only the homolog to the *Synechocystis* FtsH 4 isoform, it is unlikely that FtsH 4 is directly involved in removal of damaged PSII proteins in MIT 9313. It is tempting to speculate, given the large pool of PSI in MIT 9313 relative to PSII, that FtsH 4 homohexamers may be involved in the assembly or maintenance of PSI.

## Conclusions

We examined the PSII and FtsH protein levels, PSII activity and *ftsH* expression levels in three different picocyanobacterial strains grown under low and higher light levels. It is clear that these strains, which are adapted to different ecological niches, have very different strategies to cope with damage of their PSII reaction centres caused by high light. We conclude that the lack of capacity to cope with even transient exposure to high light levels in the low light adapted *Prochlorococcus* strain MIT 9313 is a result of the organism’s inability to rapidly induce the expression of the FtsH isoforms necessary for removal of damaged PsbA from the reaction centre. It is important to note that this strain has the genes necessary for the production of FtsH 2/3 hexamers, but when grown at 30 μmol photons m^-2^ s^-1^ it does not express these isoforms to any extent. The measurable rates of PSII repair and PsbA removal and the higher FtsH hexamer to PsbA ratio for MIT 9313 grown at 90 μmol photons m^-2^ s^-1^ suggests that when the cells have been transitioned gradually to the higher light level over a period of generations, they are capable of FtsH 2/3 synthesis even though they lack the ability to induce that capacity over a short period of 90 minutes as measured here.

The lack of capacity to remove PsbA damaged by photoinactivation, that results from the weak expression of the FtsH isoforms necessary for this function, constrains MIT 9313 to a regimen of low light growth. The strain growing under these conditions is unable to take advantage photochemically of transient exposure to high light, and appears to simply survive the exposure and recover (or not) in a subsequent period of darkness. We show that this strain does increase the rate of oxygen evolution per remaining PSII when exposed to high light, which would confer some mitigation to photoinhibition. This strain will not grow when cultured under low blue light irradiance, whereas it can grow under continuous orange/red light of the same irradiance (Campbell & Prasil, personal communication). Since blue light photons lead to photoinactivation in *Prochlorococcus* to a much greater extent than do red light photons [28], and the wavelength of light that penetrates deep into the euphotic zone is in the blue/green range, we infer that *Prochlorococcus* MIT 9313 may rely on a period of darkness for PSII repair as has been shown for marine diatoms [67].

Soitamo et al [29] have shown that oxidative stress may play a role in the collapse of *Prochlorococcus* cultures (including MIT 9313) when shifted to high light. They showed that when grown under hypoxic conditions these strains did not collapse. With MIT 9313, from the LLVI clade, originating from 150 m deep in the South Atlantic [68] it possible that a low oxygen environment has a protective effect that allows growth [69]. At low irradiance (18 μmol photons m^-2^ s^-1^) Partensky et al showed that picocyanobacterial cells consume more oxygen than they evolve, and that MIT 9313 consumes the most of the strains examined [23]. Despite these metabolic constraints, MIT 9313 grows well at 30 μmol photons m^-2^ s^-1^ (with a 12:12 light cycle) giving growth rates greater than those observed for MED 4 and even WH 8102 under the same conditions [22,70]. Clearly, there are advantages to the strategies employed for growth under low light. Taken together these findings suggest that MIT 9313 prioritizes other cellular processes that can occur at very low light levels, over linear photosynthesis starting at PSII, and the damage associated with that process.

## Supporting information

S1 Table Oxygen evolution per PSII per second before and after high light treatment, in O_2_ PSII^-1^ s^-1^. (DOCX)
