# Supplementary material for: Strain specific differences in rates of Photosystem II repair in picocyanobacteria correlate to differences in FtsH protein levels and isoform expression patterns
Source: PLoS One. 2018 Dec 19;13(12):e0209115. doi: 10.1371/journal.pone.0209115 (PMC6300248; doi:10.1371/journal.pone.0209115)
Supplement: S1 Table — (DOCX) [file pone.0209115.s001.docx]

**S1 Table:** Oxygen Evolution per PSII per second before and after high light treatment, in O_2_ PSII^-1^ s^-1^.

| Strain | Growth Light | Mean oxygen evolution per PSII per s | Standard Deviation | Count | *p* |
| --- | --- | --- | --- | --- | --- |
| MIT 9313 | 30 | 11.0 | 2.02 | 8 | 0.0155 |
| after |  | 21.1 | 8.96 | 8 |  |
| MIT 9313 | 90 | 17.5 | 2.32 | 14 | 0.0507 |
| after |  | 25.9 | 10.83 | 14 |  |
| MED 4 | 30 | 12.6 | 3.11 | 8 | 0.0593 |
| after |  | 16.8 | 4.82 | 8 |  |
| MED 4 | 260 | 14.9 | 2.12 | 4 | 0.4468 |
| after |  | 18.5 | 8.03 | 4 |  |
| WH 8102 | 30 | 22.6 | 9.45 | 10 | 0.0684 |
| after |  | 40.4 | 26.33 | 10 |  |
| WH 8102 | 260 | 34.2 | 17.57 | 4 | 0.4856 |
| after |  | 27.1 | 5.73 | 4 |  |

T-test result between start and end of high light treatment included.
